# A New Career for Women

**Published:** 1913-03-01

**Authors:** L. V. Haughton, H. A. Alsop, A. C. Gibson, E. M. Musson, M. S. Rundle, A. E. Windsor, Kate C. House, M. E. Robertson, May C. Scott, Florence Hazell, Chas. E. Hecht

**Affiliations:** Matron, Guy's Hospital (Chairman, Matrons' Committee); 178 St. Stephen's House, Westminster; Matron, Kensington Infirmary; 178 St. Stephen's House, Westminster; Ex-Matron, Birmingham Infirmary; 178 St. Stephen's House, Westminster; Matron, Birmingham General Hospital; 178 St. Stephen's House, Westminster; Matron, Royal Hospital for Diseases of the Chest, late Isla Stewart Scholar, Teachers' College, Columbia University; 178 St. Stephen's House, Westminster; trained nurse; 178 St. Stephen's House, Westminster; wife of Housemaster, Malvern College; 178 St. Stephen's House, Westminster; Headmistress, Christ's Hospital; 178 St. Stephen's House, Westminster; Headmistress, Godstone School; 178 St. Stephen's House, Westminster; formerly Lecturer on Domestic Economy and Hygiene; 178 St. Stephen's House, Westminster; Secretary, to whom all communications should be addressed. 178 St. Stephen's House, Westminster


*To the Editor of* The Hospital.

### A New Career for Women.

*To the Editor of* The Hospital.

Sir,—"The work of feeding in institutions ought," said a speaker at the Guildhall School Conference in May last, "to be regarded as a profession in itself, and should be studied from every standpoint." Two months later a representative from Toronto University informed her fellow-members of the Congress of Universities of the Empire that a new career had been found for the girl graduates in home science—they were becoming dieticians in the public hospitals. The same remark applies, in a much wider sense, to the United States, where such a profession, with the training its existence implies, has been open to women for a generation or more.

While we are far from undervaluing the training given in this country in domestic science or housecraft, still less oblivious of recent promising developments, it must be generally admitted that, mainly through lack of any demand, systematic training in institutional housekeeping, both practical and theoretical, on a scale comparable to that supplied at Teachers' College, Columbia University, and, in a greater or less degree, at many other American and Canadian universities, colleges, and training schools, has not been obtainable. The need, however, for highly trained and duly qualified women as housekeepers or cooks, as essential to effective reform, was insisted upon by numerous speakers at both the Conference of Matrons of Hospitals and Similar Institutions in 1910 and at the Conference on Diet and Hygiene in Public, Secondary, and Private Schools a year and a half later. It is certain to be urged again at this year's Conference on Diet and Hygiene in Public Elementary Schools and in Public and Philanthropic Institutions for Children and Adolescents.

Impressed with the urgency of the subject, the Matrons' and Schools Committees of the National Food Reform Association have appointed the undersigned as a Joint Committee, with instructions to bring the matter before the university and other colleges and training schools, as well as the public generally. We are confident that these institutions will not be backward in offering facilities to those desirous of entering this new field of work for women. If desired, we are prepared to co-operate further.

In connection with such developments the Association will, it may be hoped, come to be increasingly regarded as a link between duly qualified women and those seeking their services.—Yours faithfully,

L. V. Haughton, Matron, Guy's Hospital (Chairman, Matrons' Committee); H. A. Alsop, Matron, Kensington Infirmary; A. C. Gibson, Ex-Matron, Birmingham Infirmary; E. M. Musson, Matron, Birmingham General Hospital; M. S. Rundle, Matron, Royal Hospital for Diseases of the Chest, late Isla Stewart Scholar, Teachers' College, Columbia University; A. E. Windsor, trained nurse; Kate C. House, wife of Housemaster, Malvern College; M. E. Robertson, Headmistress, Christ's Hospital; May C. Scott, Headmistress, Godstone School; Florence Hazell, formerly Lecturer on Domestic Economy and Hygiene; Chas. E. Hecht, Secretary, to whom all communications should be addressed.

178 St. Stephen's House, Westminster,
February 22, 1913.

